# Mouse mucosal-associated invariant T cell receptor recognition of MR1 presenting the vitamin B metabolite, 5-(2-oxopropylideneamino)-6-d-ribitylaminouracil

**DOI:** 10.1016/j.jbc.2024.107229

**Published:** 2024-03-25

**Authors:** Lisa Ciacchi, Jeffrey Y.W. Mak, Jeremy P. Le, David P. Fairlie, James McCluskey, Alexandra J. Corbett, Jamie Rossjohn, Wael Awad

**Affiliations:** 1 Infection and Immunity Program, Department of Biochemistry and Molecular Biology, Biomedicine Discovery Institute, Monash University, Clayton, Victoria, Australia; 2 Centre for Chemistry and Drug Discovery and ARC Centre of Excellence for Innovations in Peptide and Protein Science, Institute for Molecular Bioscience, University of Queensland, Brisbane, Queensland, Australia; 3 Department of Microbiology and Immunology, Peter Doherty Institute for Infection and Immunity, The University of Melbourne, Melbourne, Victoria, Australia; 4 Institute of Infection and Immunity, School of Medicine, Cardiff University, Cardiff, UK

**Keywords:** antigen presentation, MHC-related molecule (MR1), mouse MAIT TCR, 5-OP-RU, protein structure

## Abstract

Mucosal-associated invariant T (MAIT) cells can elicit immune responses against riboflavin-based antigens presented by the evolutionary conserved MHC class I related protein, MR1. While we have an understanding of the structural basis of human MAIT cell receptor (TCR) recognition of human MR1 presenting a variety of ligands, how the semi-invariant mouse MAIT TCR binds mouse MR1-ligand remains unknown. Here, we determine the crystal structures of 2 mouse TRAV1-TRBV13-2^+^ MAIT TCR-MR1-5-OP-RU ternary complexes, whose TCRs differ only in the composition of their CDR3β loops. These mouse MAIT TCRs mediate high affinity interactions with mouse MR1-5-OP-RU and cross-recognize human MR1-5-OP-RU. Similarly, a human MAIT TCR could bind mouse MR1-5-OP-RU with high affinity. This cross-species recognition indicates the evolutionary conserved nature of this MAIT TCR–MR1 axis. Comparing crystal structures of the mouse *versus* human MAIT TCR-MR1-5-OP-RU complexes provides structural insight into the conserved nature of this MAIT TCR–MR1 interaction and conserved specificity for the microbial antigens, whereby key germline-encoded interactions required for MAIT activation are maintained. This is an important consideration for the development of MAIT cell-based therapeutics that will rely on preclinical mouse models of disease.

The highly conserved major histocompatibility complex (MHC) related protein-1 molecule, MR1, can capture and present a range of small molecule metabolites to innate-like T cells known as mucosal-associated invariant T (MAIT) cells, as well as diverse MR1-reactive αβ and γδ T cells (1, 2, 3, 4, 5). Several MR1 ligands have been identified, including the vitamin B9-based compound, 6-formylpterin (6-FP) and its synthetic derivative, acetyl-6-formylpterin (Ac-6-FP), which do not stimulate most MAIT cells (3, 6, 7). Vitamin B2 (riboflavin)-derived metabolites produced by microbes, including commensal and pathogenic bacteria or yeasts, have been described as foreign antigens presented by MR1. These include the highly potent MAIT-activating ribitylpyrimidine antigens, 5-(2-oxoethylideneamino)-6-D-ribitylaminouracil and 5-(2-oxopropylideneamino)-6-D-ribitylaminouracil (5-OP-RU), the latter being the most potent MAIT cell antigen known to date (1, 3, 8), as well as more thermodynamically stable bicyclic compounds termed ribityllumazines, such as 7-hydroxy-6-methyl-8-D-ribityl lumazine (RL-6-Me-7-OH) (1). MR1 can also accommodate a diverse panel of chemical scaffolds such as diet-derived molecules, vanillin and ethylvanillin (9); drugs, drug metabolites and drug-like molecules, (10, 11) and host-tissue–derived endogenous metabolites such as cholic acid 7-sulfate (12). To date, all identified MR1-bound antigens are sequestered within the aromatic A′ pocket of MR1, yet only compounds, including 6-FP and 5-OP-RU, that possess a reactive aldehyde or ketone functional group are known to form a stabilizing covalent imine (Schiff base) bond with lysine 43 (K43) of MR1 (1, 7, 10, 13, 14).

The MR1–MAIT cell axis is highly conserved across ∼150 million years of mammalian evolution, with ∼90% sequence identity between the α1 and α2 domains in mouse and human MR1 (15). This implies that a strong selection pressure exists to maintain a conserved population of MR1-responsive MAIT cells across mammalian species (16, 17). The MAIT cell population is characterized by the expression of a semi-invariant αβ T cell receptor (TCR) where the α-chain is composed of TRAV1-2^+^TRAJ33/12/20^+^ in humans and of orthologous TRAV1^+^ TRAJ33^+^ in mice (18, 19, 20, 21). While the TCR β-chain can be diverse, there is a preference for TRBV6/20 in humans and TRBV13/19 in mice (18, 22, 23, 24). Structural studies reveal that the capacity of the 5-OP-RU agonist to activate human MAIT cells is due to the formation of a structural motif termed an *‘interaction triad’* between the hydroxyl group of the conserved MAIT TCR TRAV1-2 CDR3α tyrosine 95 (Y95) residue, the ribityl moiety of 5-OP-RU, and tyrosine 152 (Y152) of MR1 (1, 6, 14).

Studies have been performed on TRAV1^+^ (Vα19) transgenic mice in an effort to functionally characterize mouse MAIT cells *in vivo* (18, 25, 26, 27, 28). Previously, mouse MR1 tetramers were used to isolate MR1-5-OP-RU tetramer^+^ TRBV13-2^+^ stomach MAIT cells from C57BL/6 mice primed with the live-attenuated vaccine strain, *Salmonella* *Typhimurium* BRD509 and chronically infected with *Helicobacter pylori* (18). However, the structural basis underpinning mouse MAIT TCR recognition of MR1-5-OP-RU is unknown, yet important to determine in the context of development of MAIT cell centric therapeutics that will likely depend on preclinical mouse models of disease.

To gain insight into the structural requirements for mouse MAIT TCR-MR1-Ag recognition, here we determine the crystal structures of two mouse MAIT TRBV13-2^+^ TCRs (M2A and M2B) (10, 18) which was identified from Vα19 transgenic mice (Vα19, paired with endogenous TCRβ chain) (10, 29) in complex with mouse MR1-5-OP-RU. These mouse MAIT TCRs adopt a similar molecular footprint atop mouse MR1-5-OP-RU, as in human MAIT TCR-MR1-5-OP-RU complexes (1, 14) and exhibited species cross-reactivity towards human MR1-5-OP-RU, albeit binding with weaker affinity for human MR1 indicating a species-specific preference for mouse MR1. Overall, this study has advanced our understanding of MAIT-MR1 co-evolution and MAIT TCR cross-reactivity between human and mouse MR1 in the context of recognition of antigens derived during microbial riboflavin biosynthesis.

## Results

### Reporter cells expressing a mouse MAIT TCR respond to 5-OP-RU

**Figure 1:**
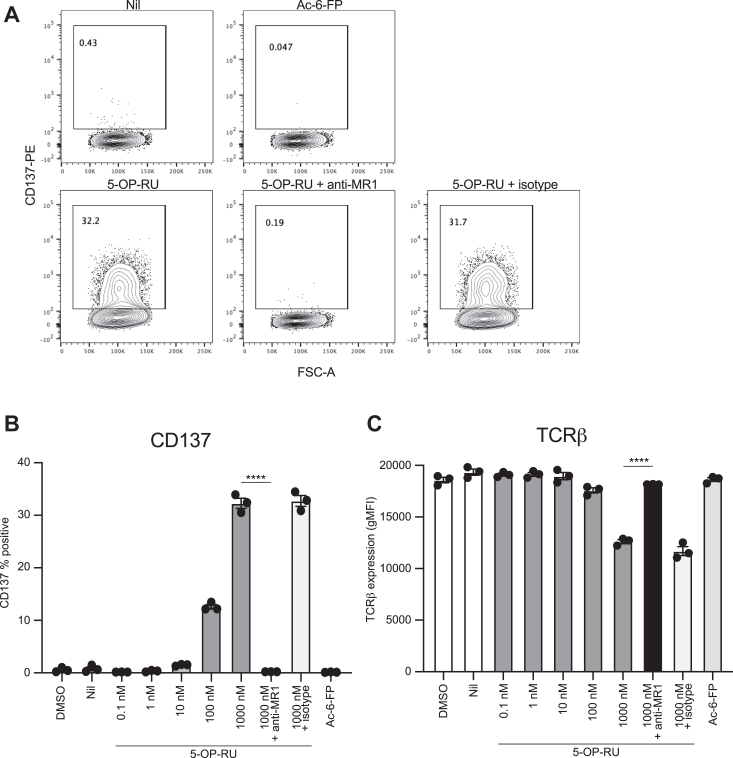
**5-OP-RU induces MR1-dependent activation of mouse MAIT reporter cells.** Representative plots (*A*) and bar graphs showing cell surface expression of CD137 (*B*) or TCRβ (*C*) on BW58.MAIT cells following overnight coculture with M12.C3.MR1 cells and indicated concentrations of 5-OP-RU, 1000 nM Ac-6-FP, or media controls. Data represent mean ± s.e.m of 3 independent repeats. ∗∗∗∗*p* < 0.0001, one-way ANOVA with Dunnett’s multiple comparisons. 5-OP-RU, 5-(2-oxopropylideneamino)-6-D-ribitylaminouracil; Ac-6-FP, acetyl-6-formylpterin; MAIT, mucosal-associated invariant T; TCR, T-cell receptor.

We have previously used human Jurkat.MAIT reporter cell lines to test reactivity to 5-OP-RU and other MR1-ligands (1, 3, 14). Therefore, we employed a similar co-culture system here. BW58 reporter cells expressing mouse CD3 and the uncharacterized M2A TCR generated by retroviral transduction (previously named “BW58.CD3.MAIT Vβ8.2 cells”) (10) were further modified to delete β2m (removing MR1-dependent T-T presentation capacity). These cells were co-cultured with the MR1-overexpressing M12.C3 cells and 5-OP-RU, and activation was measured by the upregulation of cell surface CD137 and downregulation of cell surface TCR expression. As expected, there was a dose-dependent activation in response to 5-OP-RU, but not Ac-6-FP, which could be blocked by anti-MR1 monoclonal 8F2.F9 (30), but not an isotype control mAb (Fig. 1). This assay further confirms the functionality of mouse MAIT TCRs and their reactivity towards 5-OP-RU presented by MR1.

### Mouse MAIT TCRs display differential affinities for mouse *versus* human MR1

**Figure 2:**
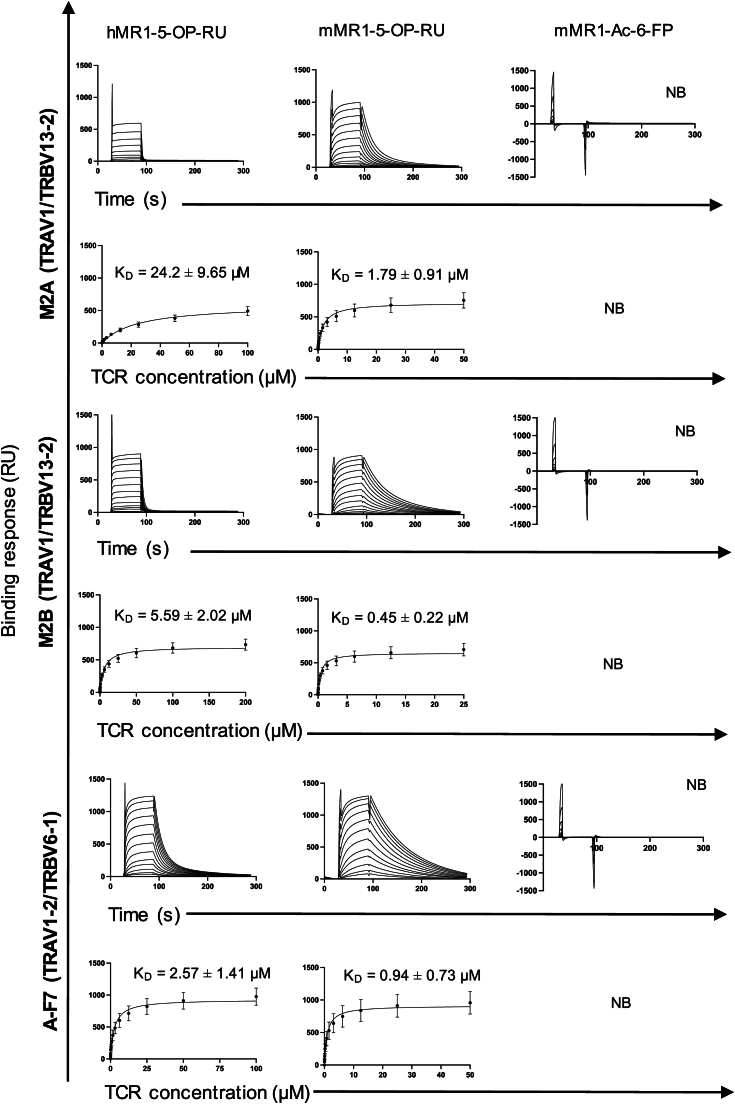
**Steady-state affinity measurements of mouse MR1-restricted TCRs.** The affinity of TCR-MR1-Ag interactions were determined using SPR, by measuring the binding of soluble mouse MAIT TCRs, M2A (*top panels*), M2B (*middle panels*), and human MAIT, A-F7 (*bottom panels*), against mouse MR1 refolded with 5-OP-RU and Ac6-FP, and human MR1 refolded with 5-OP-RU. The K_D_ values represent mean ± s.e.m values obtained from two independent experiments (n = 2) performed in technical duplicates using different batches of proteins. NB, no binding. RU, response units. The SPR sensorgrams, equilibrium curves and steady state *K*_D_ values (μM) were prepared in GraphPad Prism 10. 5-OP-RU, 5-(2-oxopropylideneamino)-6-D-ribitylaminouracil; Ac-6-FP, acetyl-6-formylpterin; MAIT, mucosal-associated invariant T; SPR, surface plasmon resonance; TCR, T-cell receptor.

To investigate how mouse MAIT TCRs bind the MR1 molecules, we expressed and purified the mouse TRAV1-TRAJ33^+^/TRBV13-2/TRBJ2-3^+^ MAIT TCRs, M2A, and M2B (18), as well as the human TRAV1-2/TRBV6-1^+^ A-F7 MAIT TCR (Table S1) as soluble recombinant proteins, then measured their binding to mouse and human MR1-ligand complexes by surface plasmon resonance (SPR). The mouse MAIT TCRs bound mouse MR1-5-OP-RU with higher affinity (ranging K_D_ ∼0.5–1.8 μM) than human MR1-5-OP-RU (K_D_ ∼ 24 μM for M2A and K_D_ ∼ 5.6 μM for M2B); neither of the mouse MAIT TCRs bound mouse MR1-Ac-6-FP (Fig. 2). The human MAIT A-F7 TCR bound human MR1-5-OP-RU with an affinity of ∼2.6 μM, consistent with published values (14) and bound mouse MR1-5-OP-RU with a 3-fold higher affinity and slower dissociation rate than human MR1-5-OP-RU (K_D_ ∼ 0.9 μM). This cross-species reactivity of the mouse and human MAIT TCRs for 5-OP-RU presented by mouse or human MR1 suggests that MAIT TCRs have co-evolved with MR1 molecules between species, suggesting evolutionary conserved nature of interactions.

### Overview of the mouse MAIT TCR-MR1-5-OP-RU ternary complexes

**Figure 3:**
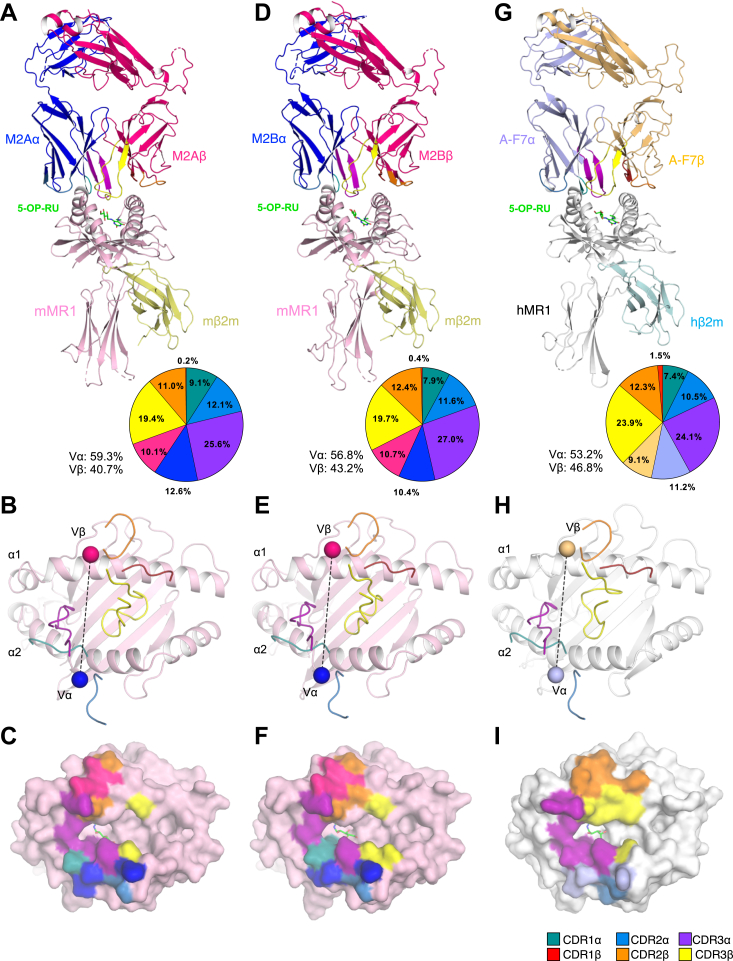
**Crystal structures of 2 mouse MAIT-MR1 complexes compared with a typical human MAIT-MR1 ternary complex.***A*–*C*, mouse M2A (TRAV1-TRBV13–2)-mMR1-5-OP-RU (*D*–*F*) M2B (TRAV1/TRBV13–2)-mMR1-5-OP-RU (*G*–*I*), A-F7 (TRAV1-2/TRBV6-1) TCR-MR1-5-OP-RU (PDB ID: 6PUC). *A*, *D*, and *G*, *top panels* are cartoon representations of the ternary complexes. For simplicity, the equivalent TCR α-chains of the mouse TCRs are colored *dark blue*; the shared mouse TCR β-chains (TRBV13–2/TRBJ2-3) are colored *dark pink.* The human TCRα A-F7 is shown in *light blue* and the human A-F7 TCRβ is shown in *light orange*. The mouse MR1 heavy chain and β2-microglobulin (β2m) molecules are colored *light pink* and *pale yellow*, respectively, whereas human MR1 heavy chain and β2m are colored *white* and *pale cyan*, respectively. The 5-OP-RU antigen is presented as *green sticks*. Pie charts represent the relative contribution of each segment of the TCRs, M2A (*A*), M2B (*D*), and A-F7 (*G*) to the buried surface area (BSA) directed against their cognate MR1-5-OP-RU complex. The corresponding complementarity determining region (CDR) loops, namely CDR1α, CDR2α, CDR3α, CDR1β, CDR2β, and CDR3β are shown in *teal, sky-blue, purple, red, orange,* and *yellow,* respectively, and the mouse TCR framework (FW) residues of the α- and β-chains are shown in *dark blue* and *dark pink,* respectively, while the human TCR FWα is colored *light orange* and FWβ in *light blue*. The *middle panels* show the CDR loops of the M2A (*B*), M2B (*E*) and A-F7 (*H*) TCRs docking onto MR1. Each docking angle is shown as a *black dashed line* connecting the center of mass of Vα with the centre of mass (COM) of Vβ which are represented as a *sphere* colored consistent with chain colors in the *upper panels*. The *lower panels* illustrate the respective mouse M2A (*C*) and M2B (*F*), and A-F7 (*I*) TCR footprints on the molecular surface of MR1-5-OP-RU. The atomic footprint is colored based on the TCR segment making contact. The surface of mouse MR1 is colored *light pink* whereas human MR1 is colored *white*. 5-OP-RU, 5-(2-oxopropylideneamino)-6-D-ribitylaminouracil; MAIT, mucosal-associated invariant T; TCR, T-cell receptor.

**Figure 4:**
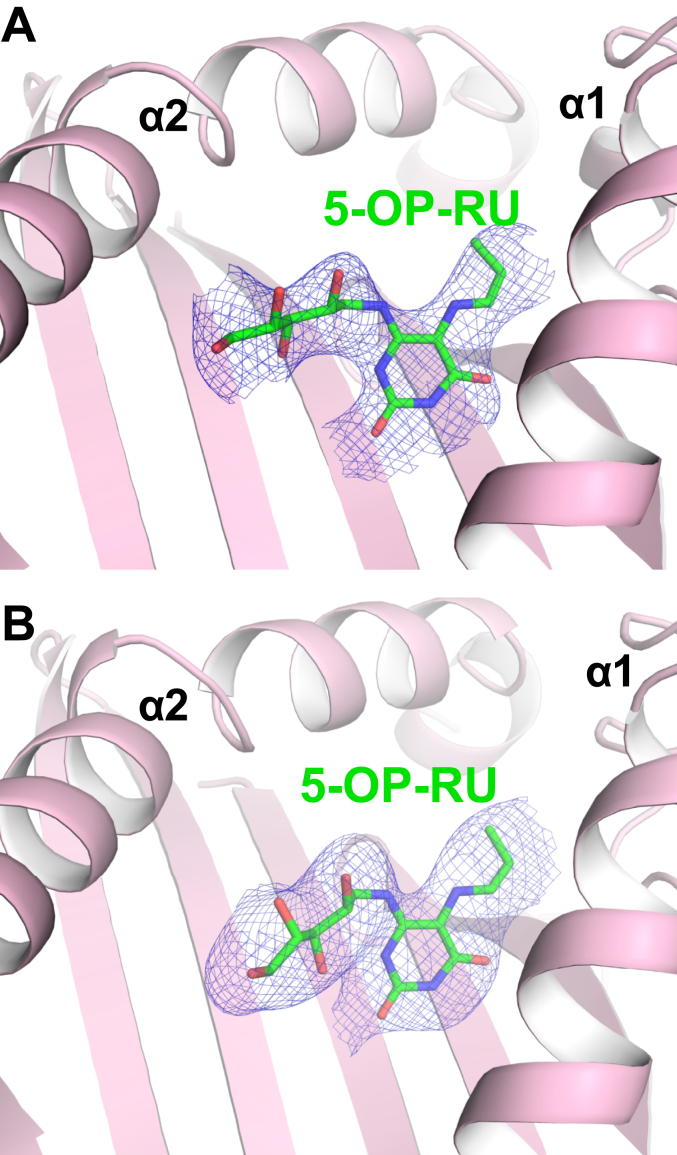
**Electron density maps of 5-OP-RU presented by TCR-docked mouse MR1.***A* and *B*, the omit maps showing unbiased Fo - Fc electron density (in *dark blue mesh*), contoured at 2 sigma, for 5-OP-RU (*green sticks*) presented by (*A* and *B*) mouse MR1 (colored *light pink*) docked by TCRs, (*A*) M2A and (*B*) M2B, respectively. 5-OP-RU, 5-(2-oxopropylideneamino)-6-D-ribitylaminouracil; TCR, T-cell receptor.

To elucidate the molecular basis of mouse MAIT TCR-MR1 recognition and compare it to human MAIT TCR-MR1 recognition, we determined the crystal structures of the M2A and M2B mouse MAIT TCRs in complexes with mouse MR1-5-OP-RU to 3.4 Å and 3.45 Å resolution, respectively (Table 1). Both mouse MAIT TCRs docked ∼88° to the main axis of the MR1-Ag–binding cleft, and the α- and β-chains were positioned atop the α2-and α1-helices of MR1, respectively (Fig. 3, *A*–*F*). The electron density at the mouse MAIT TCR/MR1 interfaces was clear enough to build 5-OP-RU and the interacting residues at the MAIT TCR-MR1 interface (Fig. 4). The buried surface area (BSA) at the interface for the mouse MAIT-MR1-5-OP-RU complexes was ∼1020 to 1040 Å^2^, which falls at the lower end of the range for TRAV1-2/TRAJ33^+^ human MAIT-MR1-5-OP-RU ternary complexes (1050–1200 Å^2^) (14, 31) (Fig. 3, *A* and *D*).

**Table 1:** Data collection and refinement statistics

	M2A TCR-mMR1-5-OP-RU	M2B TCR-mMR1-5-OP-RU
Wavelength (Å)	0.95372	0.95365
Resolution (Å)	49.22–3.40 (3.52–3.40)	43.64–3.45 (3.57–3.45)
Space group	P 2 21 21	P 2 21 21
Unit cell		
a, b, c (Å)	64.53 84.25 177.58	64.49 84.64 166.07
α, β, γ (°)	90 90 90	90 90 90
Total reflections	27,783 (2689)	24,946 (2467)
Unique reflections	13,899 (1346)	12,522 (1235)
Multiplicity	2.0 (2.0)	2.0 (2.0)
Completeness (%)	99.83 (99.48)	99.77 (100.00)
Mean I/sigma(I)	10.12 (3.37)	4.29 (1.62)
Wilson B-factor	65.51	69.09
R-merge	0.05688 (0.2499)	0.135 (0.4928)
R-meas	0.08044 (0.3533)	0.191 (0.697)
R-pim	0.05688 (0.2499)	0.135 (0.4928)
CC1/2	0.995 (0.842)	0.977 (0.602)
CC∗	0.999 (0.956)	0.994 (0.867)
R-work	0.2543 (0.2892)	0.2702 (0.3208)
R-free	0.2819 (0.3250)	0.3047 (0.3797)
Number of non-hydrogen atoms	5918	5826
Macromolecules	5882	5798
Ligands	36	28
Solvent	0	0
Protein residues	787	778
RMS bonds (Å)	0.002	0.003
RMS angles (°)	0.42	0.50
Ramachandran favored (%)	89.57	88.23
Ramachandran allowed (%)	9.39	10.85
Ramachandran outliers (%)	1.04	0.93
Rotamer outliers (%)	2.10	2.51
Average B-factor	71.32	70.38
Macromolecules	71.43	70.53
Ligands	51.91	38.73

Statistics for the highest-resolution shell are shown in parentheses.

The α-chain of both mouse TCRs contributes to the interface to a greater extent (∼60%) than the β-chain (∼40%) (Fig. 3, *A*–*D*). With respect to the Μ2A TCR, the CDR3α contributed the most to this interaction (25.6%), followed by the α-framework (FWα) region (12.6%), CDR2α (12.1%), and lastly, CDR1α (9.1%), while the CDR3β contributed ∼19.4%, followed by CDR2β (11.0%), FWβ (10.1%), and CDR1β (0.2%) (Fig. 3*B*). As anticipated, given the identical germline-encoded amino acid sequence, the relative contributions of the α- and β-chain of the M2B TCR were approximately equivalent to that of M2A TCR (Fig. 3, *A*–*F*). In contrast to the mouse MAIT TCRs, the α- and β-chains of the human A-F7 TCR contribute almost equally to the overall BSA at the MR1-5-OP-RU interface, and this is attributed to the greater involvement of the A-F7 CDR3β (23.9%) than the mouse CDR3β (Fig. 3, *G*–*I*).

### Structural basis of mouse MAIT semi-invariant TRAV1 α-chain usage

**Figure 5:**
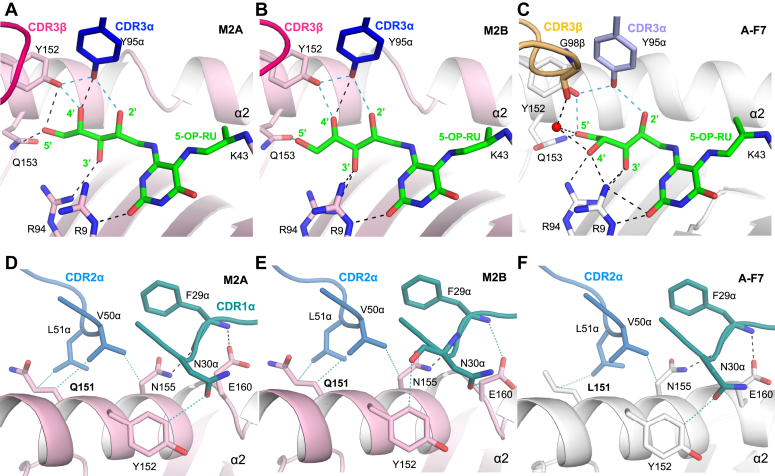
**Interface comparison of TCR α-chain of mouse MAIT *versus* human MAIT bound to relevant MR1-5-OP-RU.***A*–*C*, interactions of the CDR3α of the MAIT TCR (*A*) mouse M2A, (*B*) mouse M2B and (*C*) human A-F7, as well as MR1 residues with 5-OP-RU (colored as in Fig. 1). The MAIT TCR α and β-chains are colored as in Figure 4, *A*, *D*, and *G*. *D*–*F*, interactions of the CDR1α and CDR2α loops of MAIT TCR (*D*) mouse M2A, (*E*) mouse M2B and (*F*) human A-F7, with mouse MR1 and human MR1, respectively. Mouse MR1 and human MR1 are colored as in Figure 1. Hydrogen bonds are represented as *black dashes* (and those involved in forming the interaction triad are colored *light blue*) and van der Waals contacts are represented as *dotted teal lines*. Residues that differ between mouse MR1 and human MR1 are labeled in bold lettering. 5-OP-RU, 5-(2-oxopropylideneamino)-6-D-ribitylaminouracil; MAIT, mucosal-associated invariant T; TCR, T-cell receptor.

For both mouse TCR–MR1 complexes, 5-OP-RU bound within the A′ pocket of mouse MR1 *via* a covalent Schiff base with MR1-K43 (Fig 5, *A* and *B*). Here, the ribityl moiety of 5-OP-RU is stabilized by polar interactions with mouse MR1 residues, namely R9, R94, Y152, and Q153 (Fig. 5, *A* and *B*). Both M2A and M2B mouse MAIT TRAV1^+^ CDR3α-Y95 formed a network of hydrogen bonds, the “interaction triad”, with the 2′-OH group of the ribityl tail of 5-OP-RU and Y52 of the MR1 α1-helix, known to be essential for MAIT activation (1, 14) (Fig. 5, *A*–*C*). In addition, these mouse MAIT TCRs have a CDR1α “G-F-N” motif and CDR2α “V-L” motif that interacted with the conserved residues of MR1, including Y152, N155, W156, and E160, which are exclusive to TRAV1 (and human TRAV1-2) as found in IMGT repertoire database (Fig. 5). Here, the CDR1α F29 residues form hydrogen bonds with mouse MR1 residues, N155 and E160, and N30 contacts MR1-Y152. Further, the mouse MAIT CDR2α residues V50 and L51 bind the mouse MR1 residue, Q151 (Fig. 5, *D* and *E*; Tables S2 and S3). In common with all published human TRAV1-2^+^ MAIT-MR1-5-OP-RU complexes, the CDR1α interacts with species-conserved MR1 residues, N155, E150, and Y152; the CDR2α interacts with polymorphic human MR1 residue, L151 (Q151 in mice), and CDR3α-Y95 binds 5-OP-RU and MR1-Y152 (Figs. 5*C* and 4*F*). Altogether, the mouse MAIT semi-invariant TCR α-chain TRAV1 interactions were similar to those of the human TRAV1-2 TCR footprint on MR1.

### Role of mouse MAIT TRBV13-2 β-chains and their CDRβ loops in MR1 recognition

**Figure 6:**
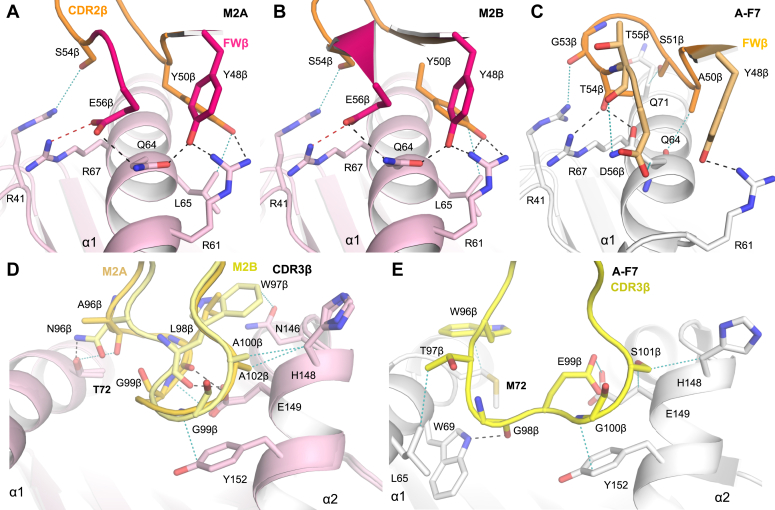
**Interface comparison of TCR beta chain of mouse MAIT TCR *versus* human MAIT TCR bound to MR1-5-OP-RU.***A*–*C*, interactions of the CDR2β (in *orange* as in Fig. 4) and framework β-chain residues of the (*A*) mouse M2A, (*B*) mouse M2B and (*C*) human A-F7 MAIT TCRs, where the FWβ residues are colored as in Figure 4, *A*, *D* and *G*. *D*, interactions of the CDR3β loops of the superimposed mouse MAIT TCRs, M2A (colored in *gold*) and M2B (colored *light yellow*). *E*, interactions of the human A-F7 CDR3β (colored in *yellow*) with MR1. Hydrogen bonds and van der Waals contacts are colored as in Figure 6. Salt bridges are represented as *red dashes*. Residues that differ between mouse MR1 and human MR1 are labeled in *bold* lettering. 5-OP-RU, 5-(2-oxopropylideneamino)-6-D-ribitylaminouracil; MAIT, mucosal-associated invariant T; TCR, T-cell receptor.

In both mouse MAIT TCR-MR1-5-OP-RU ternary complexes, the TRBV13-2^+^ CDR2β Y50 residue forms hydrogen bonds with MR1-R61, and van der Waals (vdw) contacts with MR1-L65, while the CDR2β S54 residue binds MR1-R41. In addition, the FWβ residue, E56, forms a salt-bridge with MR1-R67 and a hydrogen bond with MR1-Q64, and the FWβ Y48 forms hydrogen bonds with both mouse MR1 residues, R61 and Q64 (Fig. 6, *A* and *B*). With respect to the published structures of human MAIT TRAV1-2^+^ TCR-MR1-5-OP-RU complexes with different variable TCR β-chains (TRBV6-1/6–4/20), the CDR2-FWβ loops bind the same evolutionarily-conserved MR1 residues of R41, Q64, R67, and Q71 (Fig. 6*C*) (2, 6).

Despite the CDR3β loop of M2A having two additional amino acids than the M2B TCR, the amino acids are largely featureless in the M2A TCR CDR3β loop, where the main chain of residues, A96, L98, and G99 bind MR1. In common with the M2A TCR, the CDR3β loop of the M2B TCR binds the same mouse MR1 residues, T72, H148, and E149 with the exception of the M2B TCR also contacting MR1-N146 and Y152 (Fig. 6*D*). In the human A-F7 TCR-MR1-5-OP-RU complex, the CDR3β-W96, which is positionally equivalent to the mouse MAIT residues (A/N96), also interacts with the polymorphic MR1 residue at position 72 (*i.e.*, M72). Like the mouse MAIT TCRs, the A-F7 TCR CDR3β binds common non-polymorphic MR1 residues, Y152, H148, and E149. Additionally, the human A-F7 TCR CDR3β-T97 and G98 residues form additional contacts with MR1 α1-helix residues, L65 and W69, respectively (Fig. 6*E*).

**Figure 7:**
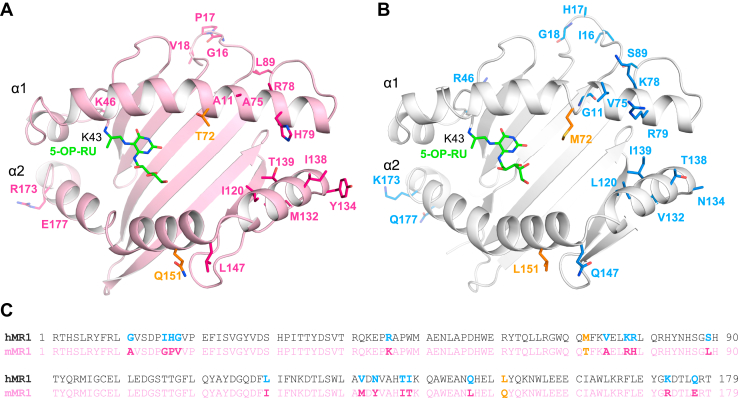
**Polymorphic residues in TCR-docked mouse *versus* human MR1-5-OP-RU molecules.** The heavy chain (α1, α2 and α3 domains) (*light pink*) of the mouse MR1 is non-covalently attached to mouse β2m (*light yellow*). The heavy chain (*white*) of the human MR1 is non-covalently attached to human β2m (*pale cyan*). 5-OP-RU is shown as *green sticks*. *A* and *B*, polymorphisms within α1-α2 domains of mouse MR1 (docked by M2A TCR) (*dark pink sticks*) (*B*) and human MR1 (docked by A-F7 TCR) (PDB: 6PUC) (*blue sticks*). Polymorphic MR1 residues contacted by TCR (*orange sticks*) (*A* and *B*) Lysine 43 forms a covalent Schiff base bond with 5-OP-RU and 6-FP in the A′ pocket of mouse MR1 (*A*) and human MR1 (*B*), respectively. *C*, alignment of residues 1 to 197 using Clustal Omega (1.2.4) of human and mouse MR1 sequences, with polymorphisms shown in bold. 6-FP, 6-formylpterin; 5-OP-RU, 5-(2-oxopropylideneamino)-6-D-ribitylaminouracil; TCR, T-cell receptor.

Taken together, these structural data revealed that mouse MAIT TCRs can engage all non-polymorphic MR1 residues *via* their germline-encoded CDR1α, CDR2α, CDR3α, CDR2β, FWβ, and non-templated CDR3β loops. Despite there being a total of 19 MR1 polymorphic differences between the α1-α2 domains in mouse and human MR1 (Fig. 7), only two of those polymorphisms at position 151 and 72 are contacted by the mouse TRAV1^+^ and human TRAV1-2^+^ MAIT TCRs, *via* their germline-encoded CDR2α and hypervariable CDR3β loops (2, 6).

## Discussion

MAIT cells have been implicated in a range of immune settings, including bacterial and viral infections, autoimmune diseases, tissue repair, and cancer, which makes them attractive vaccine targets for modulating the immune responses (13, 32, 33). Central to MAIT-MR1–mediated immunity is the recognition of the riboflavin-biosynthesis metabolite, 5-OP-RU, displayed by MR1 to a TCR on MAIT cells. Activation of MAIT cells relies on engagement of the evolutionarily conserved MAIT CDR3α-Y95 residue with 5-OP-RU and the species-conserved Y152 residue of MR1 (1, 14). Whereas human MAIT TCR-MR1–ligand interactions are now well defined, elucidating mouse MAIT TCR-MR1 binding has not advanced. Several studies have used transgenic mice expressing only the invariant orthologous TRAV1^+^ (Vα19) chain that defines mouse MAIT cells, to allow the functional characterization of MR1-restricted TRAV1^+^ T cells in the contexts of T cell development (34, 35), microbial infection (24), and autoimmune diseases (36, 37).

To examine how mouse TCRs can bind to MR1, we have analyzed two mouse MR1-5-OP-RU reactive MAIT TCRs; one from TRAV1 transgenic mice (paired with endogenous Vβ chain) and the other from WT mice, identified in the context of chronic *H. pylori* infection (18), named M2A and M2B, respectively. BW58 reporter cells expressing the M2A TCR were stimulated by 5-OP-RU presented by mouse MR1 expressing antigen presenting cells in an MR1-dependent manner and did not recognize MR1 Ac-6-FP. The affinity data revealed that these mouse MAIT TCRs exhibit high affinity for the cognate mouse MR1-5-OP-RU (M2A: K_D_ ∼1.79 μM; M2B: K_D_ ∼0.45 μM) yet have a markedly lower affinity for human MR1-5-OP-RU (by ∼13-fold for M2A: 24.2 μM; by ∼12-fold for M2B: 5.59 μM), and the dissociation of the complex is slower than for mouse MR1-5-OP-RU. Intriguingly, the human A-F7 MAIT TCR also exhibited a higher affinity for mouse MR1-5-OP-RU than its cognate human MR1-5-OP-RU. Moreover, this disparity in affinity values of the M2A, M2B, and A-F7 TCRs for mouse *versus* human MR1-5-OP-RU is likely due to the stronger engagement of TCRs with mouse-specific residues of MR1, such as the polar Q151 and T72 residues, in the antigen-binding cleft.

Previous functional studies have shown that the inability of human MR1 to activate a mouse MAIT hybridoma was due to differences in the residue at position 151 of MR1. The importance of Q151 residue for the activation of mouse MAIT cells and, surprisingly, human MAIT cells was confirmed through the use of WT3 cells presenting human MR1 with L151Q mutation. Structural comparison of human A-F7 TCR-MR1-5-OP-RU with the two mouse MAIT TCR-MR1-5-OP-RU complexes solved in this study revealed that the human TRAV1-2 and mouse TRAV1-exclusive CDR2α V-L motif interacts with L151/Q151, respectively (17). Based on both the structural and affinity data obtained in this study, it can be speculated that the reason the mouse MAIT TCRs and human A-F7 TCR could bind mouse MR1-5-OP-RU at higher affinity than human MR1-5-OP-RU is due to the presence of the essential activating Q151 residue in MR1, which is contacted by the shared CDR2α motif between mice and humans. This report also reveals key structural requirements for particular germline-encoded residues in mouse MAIT CDR1α (*i.e.*, the TRAV1-2/TRAV1-unique “G-F-N” motif), CDR2α (*i.e.*, the shared TRAV1-2/TRAV1-“V-L” motif), CDR3α (the TRAJ33-exclusive “S-N-Y-Q” motif), and TRBV13-associated residues from CDR2-FWβ, which explains the preferred gene usage of mouse MR1-restricted TRAV1^+^/TRBV13^+^ MAIT cells in the context of infection with riboflavin-producing bacteria.

This study highlights the evolutionary conserved nature of the MAIT–MR1 interaction across mice and humans. Further, the MR1 molecules of non-human primates (NHPs), including macaques, is more closely related to that of human MR1 (∼95% sequence similarity), where MAIT cell levels are maintained at frequencies similar to that of humans (38). Comparison of MR1 α1 and α2 domains from a range of NHP species with that of human MR1 revealed all 12 previously described human MR1 residues that bind 5-OP-RU are conserved (39). Despite this conservation, cross-reactivity of human MR1 tetramers was diminished when used to isolate macaque MAIT cells (38, 39). This is due to the presence of three amino acid substitutions at positions 72, 151, and 159, in all NHPs excluding chimpanzees, which were described as contact residues for human MAIT TCRs (39). Given these human MAIT TCR-contact residues are retained in chimpanzee MR1, the phenotype and functional properties of their MAIT cells are likely to be very similar to their human counterparts.

This cross-species reactivity is also observed in the context of type I natural killer T cell (NKT) recognition of lipid-based Ags presented by nonclassical MHC class-I molecule, CD1d (40). Crystal structures of human and mouse NKT TCRs in complex with CD1d-lipid Ags reveal the highly conserved docking mode dominantly mediated by the invariant NKT TCR α-chain, with the CDR1α loop binding CD1d, and CDR3α loop (as is the case in MAIT-MR1 complexes), playing a central role in contacting both the antigen-presenting molecule and the ligand (41, 42). Nevertheless, fine-specificity differences between mouse and human NKT TCRs towards CD1d-lipid can occur, which has made development of α-GalCer–related therapeutics more challenging (43). Whether there are fine specificity differences between human and mouse MAIT TCR responses towards MR1 ligands remains to be established. Such considerations are crucial for understanding the physiology of MAIT cells and will be critically important for rationally developing human-based MAIT cell therapeutics that will likely depend on the use of pre-clinical mouse models.

## Experimental procedures

### MR1-restricted ligands

Ac-6-FP (Cat. No. 11.418) was synthesized by Schircks Laboratories. Methylglyoxal was purchased from Sigma-Aldrich. 5-A-RU and 5-OP-RU were synthesized as previously described (44).

### Cell lines and culture

BW58.CD3.MAIT.TCR Vβ8.2 cells (“BW58.MAIT cells”), which express the M2A TCR, have been previously described (10). These cells were further modified here to delete β2m using CRISPR and re-cloned by flow cytometry sorting to select for high reactivity to 5-OP-RU. M12.C3.MR1 cells have been described previously (10). Both cell lines were cultured in RPMI-1640 media containing 10% fetal calf serum and passaged using 2 mM EDTA.

### Activation assay

BW58.MAIT and M12.C3.MR1 cells (10^5^ each) were co-cultured in 96-well U-bottom plates in the presence of 5-OP-RU, Ac-6-FP, or media controls. For MR1 blocking anti-MR1 mAb 8F2.F9 (30) or isotype control, MHC I reactive mAb W6/32, purified in-house, were added at 40 μg/ml for 1 h prior to addition of compounds. After overnight incubation, cells were stained with live/dead FVD-e780 (Invitrogen, 1:1000) in PBS, blocked with 2.4G2 hybridoma supernatant for 15 min at room temperature, then stained with biotinylated anti-MR1 (8F2.F9, 5.8 μg/ml), CD137-PE (17B5, Biolegend, 1:200), and TCRβ-APC (H57-597, Biolegend, 1:200), followed by SA-BV421 (Biolegend, 1:1000) in PBS containing 2% fetal calf serum and 2 mM EDTA, before acquisition on a BD Fortessa flow cytometer using Diva Software (https://www.bdbiosciences.com/en-au/products/software/instrument-software). Analysis was performed using FlowJo software (https://www.flowjo.com/) (v10) and activation of BW58.MAIT cells measured by an increase in CD137 and decrease in TCRβ surface expression.

### Protein expression, refolding, and purification of soluble MR1-Ag and MAIT TCRs

Soluble human A-F7 MAIT TCR was refolded from inclusion bodies as described previously (6, 8). For mouse MAIT TCRs, 120 mg of each TCR α- and β-chain were added in 1 L refold buffer consisting of 0.1 M Tris–HCl pH 8.5, 2.5 M urea, 0.8 M L-arginine, 2 mM EDTA, 2.5 mM oxidized glutathione, and 20 mM reduced glutathione for 3 days at 4 °C. Both the human and mouse versions of MR1-β2m were refolded in the presence of methylglyoxal and 5-A-RU (for 5-OP-RU formation) (as previously described (1, 3)). The refolded MAIT TCR or MR1-β2m-ligand complex were dialyzed against 10 mM Tris–HCl pH 8.0 and then purified by DEAE (GE Healthcare) anion exchange chromatography, followed by size-exclusion chromatography (Superdex 200, GE Healthcare) and lastly, anion exchange (HiTrap-Q HP) chromatography, as previously described (3). Protein purity was determined by SDS-PAGE, and concentrations were calculated from absorbance values at A_280nm_ using a NanoDrop spectrophotometer.

### SPR measurements

All SPR experiments were conducted in duplicate (at least two independent experiments) at 25 °C on a BIAcore T200 instrument using HBS buffer: 10 mM HEPES–HCl pH 7.5, 150 mM NaCl, and 0.005% surfactant P20 supplied by the manufacturer (GE Healthcare), as described previously (6). Biotinylated C-terminally cysteine-tagged human and mouse MR1-Ag complexes (generated as described previously in (8) were immobilized on streptavidin sensor chips with a surface density of ∼2000 response units (RU). Each streptavidin-chip comprises four flow cells; one was loaded with mouse MR1-5-OP-RU, the other was loaded with human MR1-5-OP-RU, and the last cell was left empty to subtract for nonspecific binding. Various concentrations of MAIT TCRs (serially diluted from 200 μM) were injected over the chip at a rate of 5 μl/min. Data were plotted using the 1:1 Langmuir-binding model in Prism version 10 (GraphPad) software (https://www.graphpad.com/features).

### Crystallization, structure determination, and refinement

For ternary complexation, purified mouse TCR was mixed with mouse MR1-β2m-5-OP-RU in a 1:1 M ratio at a concentration of ∼4 to 6 mg/ml and incubated on ice for 1 h. Crystals were obtained *via* hanging drop vapor diffusion method. Crystals of ternary mouse MAIT TCRs in complex with mouse MR1-5-OP-RU formed in 0.1 M Bis-Tris Propane (pH 7.5–8.5), 16 to 22% PEG 3350, and 0.2 M sodium acetate. Before flash freezing in liquid nitrogen, crystals were soaked in reservoir solution supplemented with 10 to 15% glycerol for cryoprotection. X-ray diffraction data were collected at 100 K at the Australian Synchrotron at either MX1 or MX2 beamlines. Diffraction images were indexed, integrated, and scaled using XDS (45) and were further processed using Aimless in the CCP4 suite (46) and Phenix package (47). Phases were calculated by molecular replacement, and the initial search model for MR1 complex was human MR1 complex (PDB 4GUP), where the ligand was removed, to minimize model bias, and a A-F7 TCR (PDB ID: 4L4T) was used as a search model for the mouse MAIT TCRs. Model building was undertaken in COOT (48) accompanied by iterative rounds of refinement using Phenix.refine software (https://phenix-online.org/documentation/reference/refine_gui.html). The GradeWeb server and Phenix tools were used to build and generate ligand restraints. Validation of models was achieved using MolProbity (49) and all graphical representations were generated using the PyMOL Molecular Graphics System, version 2.5. Calculations of BSAs were achieved using CCP4 AreaIMol in the CCP4 suite (46).

## Data availability

The accession number for the atomic coordinates of ternary mouse MAIT M2A and M2B TCRs-MR1-ligand and with associated structure factors have been deposited at the protein databank (www.rcsb.org) with accession codes 8VZ9, and 8VZ8 respectively.

## Supporting information

This article contains supporting information.

## Conflict of interest

J. Y. W. M., D. P. F., J. M., A. J. C., and J. R. are named inventors on patent applications (PCT/AU2013/000742, WO2014005194) (PCT/AU2015/050148, WO2015149130) describing MR1 ligands and MR1 tetramers. All other authors declare that they have no conflicts of interests with the contents of this article.
