# Supplementary material for: Mouse mucosal-associated invariant T cell receptor recognition of MR1 presenting the vitamin B metabolite, 5-(2-oxopropylideneamino)-6-d-ribitylaminouracil
Source: J Biol Chem. 2024 Mar 25;300(5):107229. doi: 10.1016/j.jbc.2024.107229 (PMC11066510; doi:10.1016/j.jbc.2024.107229)
Supplement: Supporting Tables S1–S3 [file mmc1.docx]

**Mouse mucosal-associated invariant T cell receptor recognition of MR1 presenting the vitamin B metabolite, 5-(2-oxopropylideneamino)-6-D-ribitylaminouracil**

Lisa Ciacchi^1^, Jeffrey Y.W. Mak^2^, Jeremy P. Le^3^, David P. Fairlie^2^, James McCluskey^3^, Alexandra J. Corbett^3^, Jamie Rossjohn^1, 4#^ & Wael Awad^1#^

^1^ Infection and Immunity Program, Department of Biochemistry and Molecular Biology, Biomedicine Discovery Institute, Monash University, Clayton, Victoria 3800, Australia. ^2^ Centre for Chemistry and Drug Discovery and ARC Centre of Excellence for Innovations in Peptide and Protein Science, Institute for Molecular Bioscience, University of Queensland, Brisbane Queensland 4072, Australia. ^3^ Department of Microbiology and Immunology, Peter Doherty Institute for Infection and Immunity, The University of Melbourne, Melbourne, Victoria 3000, Australia. ^4^ Institute of Infection and Immunity, Cardiff University, School of Medicine, Heath Park, Cardiff CF14 4XN, UK

^#^ Joint senior and corresponding authors, [Jamie.rossjohn@monash.edu](mailto:Jamie.rossjohn@monash.edu), [wael.awad@monash.edu](mailto:wael.awad@monash.edu)

**SUPPORTING DATA**

**Table S1. List of TCR α- and β-chain sequences**

| **TCR** | **TRAV** | **CDR1**α | **CDR2**α | **CDR3**α | **TRAJ** | **TRBV** | **CDR1**β | **CDR2**β | **CDR3**β | **TRBJ** | **Refs** |
| --- | --- | --- | --- | --- | --- | --- | --- | --- | --- | --- | --- |
| M2A | 1 | TSGFNG | VVLDGL | CAVRDSNYQLIW | 33 | 13-2 | NNHNN | SYGAGS | CASGDAKLGVGAETLYF | 2-3 | (10) |
| M2B | 1 | TSGFNG | VVLDGL | CAVRDSNYQLIW | 33 | 13-2 | NNHNN | SYGAGS | CASGDNWGGAETLYF | 2-3 | (18) |
| A-F7 | 1-2 | TSGFNG | NVLDGL | CAVKDSNYQLIW | 33 | 6-1 | MNHNS | SASEGT | CASSVWTGEGSGELF | 2-2 | (47) |

Red text represents residues encoded by non-templated nucleotides

**Table S2. Contacts of M2A TCR with mouse MR1-5-OP-RU**

| **TCR gene** | **TCR residue** | **MR1** | **Bond type** |
| --- | --- | --- | --- |
| CDR1α | Gly28  Phe29  Phe29  Asn30 | Glu160  Asn155 & Glu160  Glu160  Tyr152 | VDW  HB  VDW  VDW |
| CDR2α | Val50  Leu51 | Gln151 & Tyr152  Gln151 | VDW  VDW |
| CDR3α | Ser93  Ser93  Asn94  Tyr95  Tyr95 | Tyr62  Trp164  Arg61, Tyr62 & Trp164  Arg61 & Tyr152  Arg61, Leu65, Tyr152 & Trp156 | HB  VDW  VDW  HB  VDW |
| α-framework | Tyr48  Arg66  Arg66  Arg66 | His148 & Tyr152  Asn155  Glu159  Asn155 & Glu159 | VDW  HB  SB  VDW |
| CDR2β | Tyr50  Tyr50  Ser54 | Arg61  Leu65  Arg41 | HB  VDW  VDW |
| CDR3β | Ala96  Leu98  Leu98  Gly99  Ala102 | Thr72  Glu149  Glu149  Glu149  His148 & Gln149 | VDW  HB  VDW  VDW  VDW |
| β-framework | Tyr48  Tyr48  Glu56  Glu56  Glu56 | Arg61 & Gln64  Gln64  Arg67  Gln64  Gln64 | HB  VDW  SB  HB  VDW |
| CDR3α | Tyr95 | **5-OP-RU**  2’-OH, 3’-OH | HB |

Atomic contacts determined via the CONTACT program of the CCP4i package suite with cut-off of 4 Å. Hydrogen bond (HB) interactions are defined as contact distances of < 3.5 Å. Van der Waals (VDW) interactions are defined as non-hydrogen bond contact distances of < 4 Å. Salt bridge (SB) interactions are defined as contact distances of < 4.5 Å.

**Table S3. Contacts of M2B TCR with mouse MR1-5-OP-RU**

| **TCR gene** | **TCR residue** | **MR1** | **Bond type** |
| --- | --- | --- | --- |
| CDR1α | Phe29  Asn30 | Asn155 & Glu160  Tyr152, Trp156 & Glu160 | HB  VDW |
| CDR2α | Val50  Leu51 | Gln151, Tyr152 & Asn155  Gln151 | VDW  VDW |
| CDR3α | Ser93  Ser93  Asn94  Tyr95  Tyr95 | Tyr62  Tyr62 & Trp164  Arg61, Tyr62 & Trp164  Arg61 & Tyr152  Arg61, Leu65 & Trp156 | HB  VDW  VDW  HB  VDW |
| α-framework | Tyr48  Arg66  Arg66 | His148 & Tyr152  Asn155  Asn155 & Glu159 | VDW  HB  VDW |
| CDR2β | Tyr50  Tyr50  Tyr50  Ser54 | Arg61  Gln64  Leu65 & Gly68  Arg41 | HB  VDW  VDW  VDW |
| CDR3β | Asn96  Trp97  Trp97  Gly99  Ala100 | Thr72  Glu149  Asn146 & Glu149  Tyr152  His148 | HB  HB  VDW  VDW  VDW |
| β-framework | Tyr48  Tyr48  Glu56  Glu56  Glu56 | Arg61 & Gln64  Arg61 & Gln64  Arg67  Gln64  Gln64 | HB  VDW  SB  HB  VDW |
| CDR3α | Tyr95 | **5-OP-RU**  2’-OH, 3’-OH | HB |

Atomic contacts determined via the CONTACT program of the CCP4i package suite with cut-off of 4 Å. Hydrogen bond (HB) interactions are defined as contact distances of < 3.5 Å. Van der Waals (VDW) interactions are defined as non-hydrogen bond contact distances of < 4 Å. Salt bridge (SB) interactions are defined as contact distances of < 4.5 Å.
